# Supplementary material for: Fibroblasts are a site of murine cytomegalovirus lytic replication and Stat1-dependent latent persistence in vivo
Source: Nat Commun. 2023 May 29;14:3087. doi: 10.1038/s41467-023-38449-x (PMC10227055; doi:10.1038/s41467-023-38449-x)
Supplement: Supplementary file 1 — Supplementary Information [file 41467_2023_38449_MOESM1_ESM.pdf]

Supplementary Figure 1

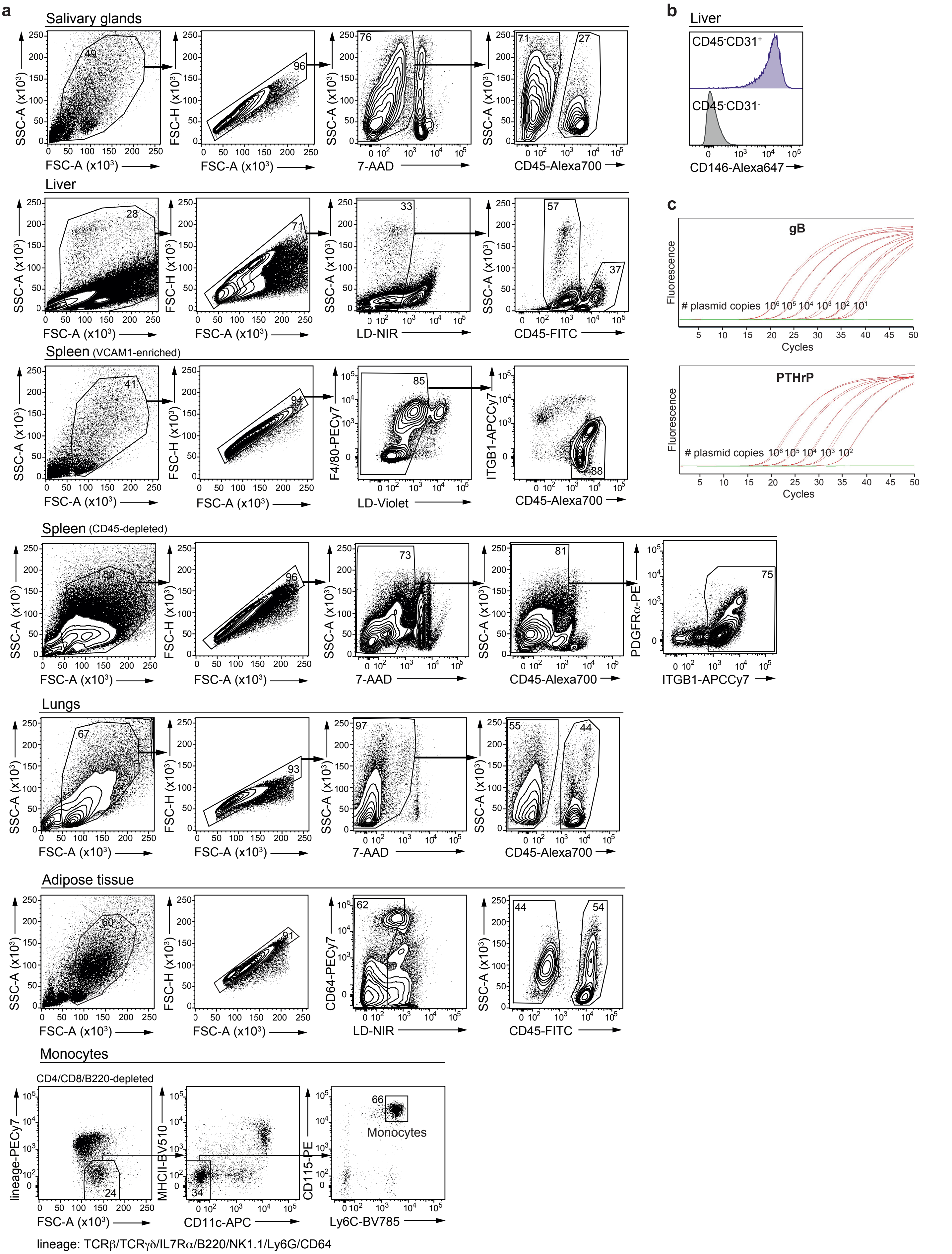

Supplementary Figure 1. Related to Figures 1-2. a Pre-gating strategy used for the sorting of the indicated cell subsets. **b** CD146 expression by liver EC. Representative plots from 2 mice. **c** Dynamic range of the qPCR assay for quantification of MCMV and mouse genome copies validated using serial dilutions of a plasmid with inserted viral *gB* and mouse *Pthrp* genomic sequences.

Supplementary Figure 2

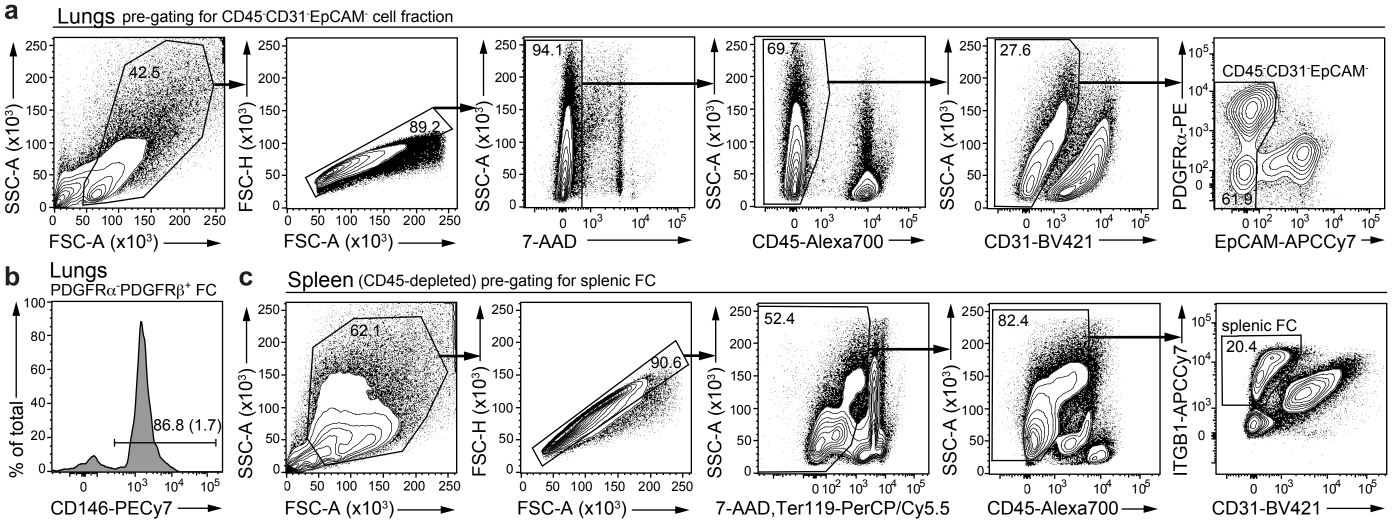

Supplementary Figure 2. Related to Figure 3. a Pre-gating strategy used for the sorting of the CD45$^-$CD31$^-$EpCAM$^-$ cell fraction from the lungs. **b** CD146 expression by lung PDGFRα$^-$PDGFRβ$^+$ FC. Shown is arithmetic mean ± SD from n = 3 biologically independent mice examined over 2 independent experiments. **c** Pre-gating strategy used for the sorting of splenic FC.

Supplementary Figure 3

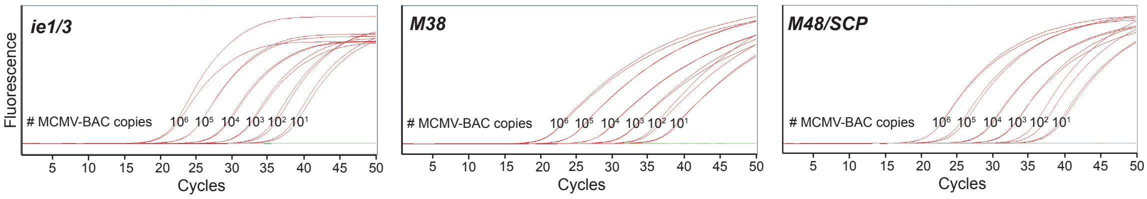

Supplementary Figure 3. Related to Table 1. Dynamic range of the qPCR assay for quantification of *ie1/3*, *M38* and *M48/SCP* cDNA copies validated using serial dilutions of MCMV BAC.

Supplementary Figure 4

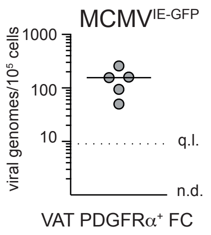

Supplementary Figure 4. Related to Figure 4. MCMV genome load in PDGFRα^+ FC purified from the VAT of mice latently infected with 10^6 PFU of MCMV$^{IE\text{-}GFP}$ administered intraperitoneally 5 months prior. Horizontal line depicts median from n = 5 biologically independent mice (depicted as symbols) examined over 2 independent experiments. q.l., quantification limit; n.d., not detected.

Supplementary Figure 5

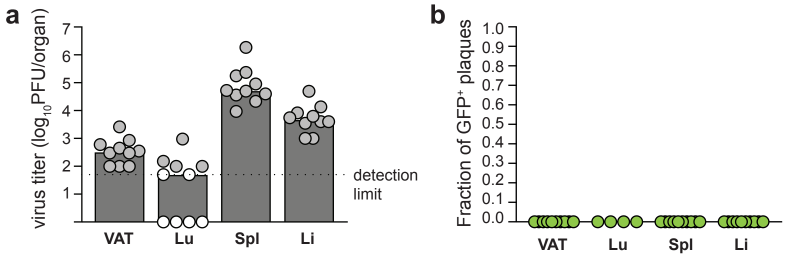

Supplementary Figure 5. Related to Figure 5. Plots show **a** median virus titer and **b** the absence of GFP$^+$ plaques following intraperitoneal infection of Cre-negative littermate control mice with MCMV$^{floxSTOP\text{-}GFP}$. Circles represent samples from individual mice. **a** Grey-filled circles are samples with sufficient virus titer to enable examination of GFP$^+$ plaques. White-filled circles are samples in which virus titer was too low to enable examination of GFP$^+$ plaques.

Supplementary Figure 6

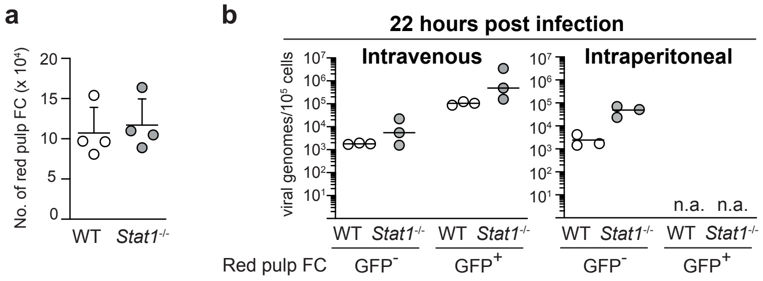

Supplementary Figure 6. Related to Figure 6. a Number of red pulp FC in 14 weeks old WT or Stat1$^{-/-}$ mice. Data are presented as the arithmetic mean ± SD of n = 4 biologically independent mice and are from 1 experiment. **b** Number of virus genomes per 100,000 of red pulp FC. Data are presented as the median of n = 3 biologically independent mice and are from 1 experiment performed for each infection route. n.a., not analysed.

Supplementary Table 1

Type of infection	Cell type	Estimated number of cells per reaction	MCMV transcript copies detected per reaction					
			ie1/3		*M38*		*M48(SCP)*	
5 months after intraperitoneal infection	Spleen PDGFRα^{+} FC	8,911	n.d.	n.d.	n.d.	n.d.	n.d.	n.d.
		9,153	n.d.	n.d.	n.d.	n.d.	n.d.	n.d.
		7,630	n.d.	n.d.	n.d.	n.d.	n.d.	n.d.
	Visceral adipose tissue PDGFRα^{+} FC	8,359	n.d.	n.d.	n.d.	n.d.	n.d.	n.d.
		7,659	n.d.	n.d.	n.d.	n.d.	n.d.	n.d.
		11,799	n.d.	n.d.	n.d.	n.d.	n.d.	n.d.
	Salivary gland PDGFRα^{+} FC	5,468	n.d.	n.d.	n.d.	n.d.	n.d.	n.d.
		6,242	n.d.	n.d.	n.d.	n.d.	n.d.	n.d.
		6,242	n.d.	n.d.	n.d.	n.d.	n.d.	n.d.
	Liver PDGFRα^{+} FC	6,224	n.d.	n.d.	n.d.	n.d.	n.d.	n.d.
		6,735	n.d.	n.d.	n.d.	n.d.	n.d.	n.d.
		6,715	n.d.	n.d.	n.d.	n.d.	n.d.	n.d.
	Liver EC	9,333	n.d.	n.d.	n.d.	n.d.	n.d.	n.d.
		6,618	n.d.	n.d.	n.d.	n.d.	n.d.	n.d.
		9,488	n.d.	n.d.	n.d.	n.d.	n.d.	n.d.
	Lung PDGFRα^{+} FC (negative control)	14,431	n.d.	n.d.	n.d.	n.d.	n.d.	n.d.
		15,234	n.d.	n.d.	n.d.	n.d.	n.d.	n.d.
		16,971	n.d.	n.d.	n.d.	n.d.	n.d.	n.d.
5 months after intranasal infection	Lung PDGFRα^{+} FC	16,971	n.d.	n.d.	n.d.	n.d.	n.d.	n.d.
		14,756	n.d.	n.d.	n.d.	n.d.	n.d.	n.d.
		17,261	n.d.	n.d.	n.d.	n.d.	n.d.	n.d.
3 months after intraperitoneal infection	Visceral adipose tissue PDGFRα^{+} FC	10,626	n.d.	n.d.	n.d.	n.d.	n.d.	n.d.
		11,751	n.d.	n.d.	n.d.	n.d.	n.d.	n.d.
		9,498	n.d.	n.d.	n.d.	n.d.	n.d.	n.d.
	Visceral adipose tissue Mϕ	10,551	n.d.	n.d.	n.d.	n.d.	n.d.	n.d.
		12,228	n.d.	n.d.	n.d.	n.d.	n.d.	n.d.
		10,975	n.d.	n.d.	n.d.	n.d.	n.d.	n.d.

n.d., not detected; MCMV, murine cytomegalovirus; FC, fibroblastic cells; Mϕ, macrophages

Supplementary Table 1. Related to Table 1. RT-qPCR of no reverse transcriptase controls prepared from the same RNA samples that were used to prepare cDNA analysed in Table 1. Each row represents a different biological replicate analysed in 2 reactions (technical duplicates) per MCMV gene. Biological replicates represent cells sorted from pooled preparations from 1 (lung, liver, salivary glands), 2 (visceral adipose tissue) or 4 (spleen) mice per replicate.

Supplementary Table 2. Antibodies

Antibody	Source	Identifier	Dilution
APC/Cy7 anti-mouse ITGB1 (clone HMβ1-1)	BioLegend	Cat# 102226, RRID: AB_2128076	1:300
AF700 anti-mouse CD45.2 (clone 104)	BioLegend	Cat# 109822, RRID: AB_493731	1:100
FITC anti-mouse CD45.2 (clone 104)	BioLegend	Cat# 109806, RRID: AB_313443	1:200
FITC anti-mouse BST1 (clone KT157)	eBioscience	Cat# MA5-17948, RRID: AB_2539332	1:50
APC anti-mouse BST1 (clone BP-3)	BioLegend	Cat# 140208, RRID: AB_10901172	1:1000
PE anti-mouse PDGFRα (clone APA5)	BioLegend	Cat# 135906, RRID: AB_1953269	1:100
APC anti-mouse PDGFRα (clone APA5)	BioLegend	Cat# 135908, RRID: AB_2043970	1:50
PE anti-mouse PDGFRβ (clone APB5)	BioLegend	Cat# 136006, RRID: AB_1953271	1:50
PE/Cy7 anti-mouse CD146 (clone ME-9F1)	BioLegend	Cat# 134714, RRID: AB_2563109	1:400
AF647 anti-mouse CD146 (clone ME-9F1)	BioLegend	Cat# 134717, RRID: AB_2721426	1:400
BV510 anti-mouse Ly6C (clone HK1.4)	BioLegend	Cat# 128033, RRID: AB_2562351	1:200
PE/Cy7 anti-mouse Ly6C (clone HK1.4)	BioLegend	Cat# 128018, RRID: AB_1732082	1:400
BV421 anti-mouse CD31 (clone MEC13.3)	BD Biosciences	Cat# 562939, RRID: AB_2665476	1:100 (SPL), other 1:400
PE/Cy7 anti-mouse CD31 (clone MEC13.3)	BioLegend	Cat# 102524, RRID: AB_2572182	1:100 (SPL), other 1:400
PE anti-mouse VCAM1 (clone 429 (MVCAM.A))	BioLegend	Cat# 105714, RRID: AB_1134164	1:100
PE/Cy7 anti-mouse F4/80 (clone BM8)	BioLegend	Cat# 123113, RRID: AB_893490	1:200
APC anti-mouse F4/80 (clone BM8)	BioLegend	Cat# 123115, RRID: AB_893493	1:200
APC/Cy7 anti-mouse EpCAM (clone G8.8)	BioLegend	Cat# 118218, RRID: AB_2098648	1:100
PerCP/Cy5.5 anti-mouse Ly6G (clone 1A8)	BioLegend	Cat# 127616, RRID: AB_1877271	1:200
PerCP/Cy5.5 anti-mouse Ly6C (clone HK1.4)	BioLegend	Cat# 128012, RRID: AB_1659241	1:200
PerCP/Cy5.5 anti-mouse Siglec-F (clone S17007L)	BioLegend	Cat# 155526, RRID: AB_2890714	1:200
PerCP/Cy5.5 anti-mouse TER-119 (clone TER-119)	BioLegend	Cat# 116228, RRID: AB_893636	1:100
PE anti-mouse Siglec-F (clone S17007L)	BioLegend	Cat# 155505, RRID: AB_2750234	1:400
BV510 anti-mouse/human CD11b (clone M1/70)	BioLegend	Cat# 101263, RRID: AB_2629529	1:200
PE/Cy7 anti-mouse CD64 (clone X54-5/7.1)	BioLegend	Cat# 139314, RRID: AB_2563904	1:200
BV510 anti-mouse CD11c (clone N418)	BioLegend	Cat# 117353, RRID: AB_2686978	1:200
PE anti-mouse CD115 (clone AFS98)	BD Biosciences	Cat# 566839, RRID: AB_2869896	1:100
BV785 anti-mouse Ly6C (clone HK1.4)	BioLegend	Cat# 128041, RRID: AB_2565852	1:500
APC anti-mouse CD11c (clone N418)	BioLegend	Cat# 117309, RRID: AB_313778	1:200
BV510 anti-mouse I-A/I-E (clone M5/114.15.2)	BioLegend	Cat# 107636, RRID: AB_2734168	1:200
FITC anti-mouse/human CD11b (clone M1/70)	BioLegend	Cat# 101205, RRID: AB_312788	1:200
PE/Cy7 anti-mouse TCR β chain (clone H57-597)	BioLegend	Cat# 109221, RRID: AB_893627	1:200
PE/Cy7 anti-mouse TCR γ/δ (clone GL3)	BioLegend	Cat# 118123, RRID: AB_11203530	1:200
PE/Cy7 anti-mouse IL-7Rα (clone S18006K)	BioLegend	Cat# 158209, RRID: AB_2922489	1:200
PE/Cy7 anti-mouse/human CD45R/B220 (clone RA3-6B2)	BioLegend	Cat# 103221, RRID: AB_313004	1:200
PE/Cy7 anti-mouse NK-1.1 (clone PK136)	BioLegend	Cat# 108713, RRID: AB_389363	1:200
PE/Cy7 anti-mouse Ly6G (clone 1A8)	BioLegend	Cat# 127618, RRID: AB_1877261	1:200
Anti-m123/IE1 (MCMV) antibody (clone IE1.01)	CapRi	Cat# HR-MCMV-12, RRID: unknown	1:200
AF647 anti-mouse IgG (H+L), $F(ab')_2$ Fragment	Cell Signaling	Cat# 4410S, RRID: unknown	1:500
