# Supplementary material for: Fibroblasts are a site of murine cytomegalovirus lytic replication and Stat1-dependent latent persistence in vivo
Source: Nat Commun. 2023 May 29;14:3087. doi: 10.1038/s41467-023-38449-x (PMC10227055; doi:10.1038/s41467-023-38449-x)
Supplement: Supplementary file 2 — Reporting Summary [file 41467_2023_38449_MOESM2_ESM.pdf]

nature portfolio

Corresponding author(s): Luka Cicin-Sain (lead contact), Katarzyna Sitnik

Last updated by author(s): Apr 14, 2023

Reporting Summary

Nature Portfolio wishes to improve the reproducibility of the work that we publish. This form provides structure for consistency and transparency in reporting. For further information on Nature Portfolio policies, see our Editorial Policies and the Editorial Policy Checklist.

Statistics

For all statistical analyses, confirm that the following items are present in the figure legend, table legend, main text, or Methods section.

n/a	Confirmed	
☐	☒	The exact sample size (*n*) for each experimental group/condition, given as a discrete number and unit of measurement
☐	☒	A statement on whether measurements were taken from distinct samples or whether the same sample was measured repeatedly
☐	☒	The statistical test(s) used AND whether they are one- or two-sided<br>*Only common tests should be described solely by name; describe more complex techniques in the Methods section.*
☒	☐	A description of all covariates tested
☐	☒	A description of any assumptions or corrections, such as tests of normality and adjustment for multiple comparisons
☐	☒	A full description of the statistical parameters including central tendency (e.g. means) or other basic estimates (e.g. regression coefficient) AND variation (e.g. standard deviation) or associated estimates of uncertainty (e.g. confidence intervals)
☐	☒	For null hypothesis testing, the test statistic (e.g. *F*, *t*, *r*) with confidence intervals, effect sizes, degrees of freedom and *P* value noted<br>*Give P values as exact values whenever suitable.*
☒	☐	For Bayesian analysis, information on the choice of priors and Markov chain Monte Carlo settings
☒	☐	For hierarchical and complex designs, identification of the appropriate level for tests and full reporting of outcomes
☒	☐	Estimates of effect sizes (e.g. Cohen's *d*, Pearson's *r*), indicating how they were calculated

Our web collection on statistics for biologists contains articles on many of the points above.

Software and code

Policy information about availability of computer code

Data collection	IncuCyte S3 (Sartorius) running IncuCyte S3 Software version v2021B<br>LightCycler 480 (Roche) running LightCycler 480 Software version 1.5.0.39<br>Zeiss LSM 980 running ZEN (blue edition) Software version 3.8.20314.48
Data analysis	FlowJo version 10 (www.flowjo.com)<br>GraphPad Prism version 9 (www.graphpad.com)<br>ZEN blue (ZEN lite) version 3.5.093.0004 (www.zeiss.com)<br>IncuCyte S3 software version v2021B (www.sartorius.com)<br>LightCycler 480 software version 1.5.0.39 (https://diagnostics.roche.com/global/en/products/instruments/lightcycler-480-ins-445.html)

For manuscripts utilizing custom algorithms or software that are central to the research but not yet described in published literature, software must be made available to editors and reviewers. We strongly encourage code deposition in a community repository (e.g. GitHub). See the Nature Portfolio guidelines for submitting code & software for further information.

Data

Policy information about availability of data

All manuscripts must include a data availability statement. This statement should provide the following information, where applicable:

- Accession codes, unique identifiers, or web links for publicly available datasets
- A description of any restrictions on data availability
- For clinical datasets or third party data, please ensure that the statement adheres to our policy

Data availability statement has been included. Source data file with associated raw data is provided with the study.

Human research participants

Policy information about studies involving human research participants and Sex and Gender in Research.

Reporting on sex and gender	This study did not use human research participants.
Population characteristics	not applicable
Recruitment	not applicable
Ethics oversight	not applicable

Note that full information on the approval of the study protocol must also be provided in the manuscript.

Field-specific reporting

Please select the one below that is the best fit for your research. If you are not sure, read the appropriate sections before making your selection.

☒ Life sciences ☐ Behavioural & social sciences ☐ Ecological, evolutionary & environmental sciences

For a reference copy of the document with all sections, see nature.com/documents/nr-reporting-summary-flat.pdf

Life sciences study design

All studies must disclose on these points even when the disclosure is negative.

Sample size	We did not perform specific power calculations but had a minimum of 3 biologically independent samples per group and all experiments were repeated at least 2 times. This was restricted by the number of mice with the correct genotype and by the scarcity of cell populations analyzed which required pooling of cell preparations from several mice to obtain one replicate.
Data exclusions	No data were excluded from the study.
Replication	Results were reproduced by at least 2 independent experiments and all repeats were successful.
Randomization	Mice were allocated to groups based on genotype.
Blinding	The investigators were not blinded to allocation during experiments as this was not deemed necessary since the type of readouts were not subjective and also to avoid/control for potential cross-contamination between the samples.

Reporting for specific materials, systems and methods

We require information from authors about some types of materials, experimental systems and methods used in many studies. Here, indicate whether each material, system or method listed is relevant to your study. If you are not sure if a list item applies to your research, read the appropriate section before selecting a response.

Materials & experimental systems

n/a	Involved in the study	
[ ]	[x]	Antibodies
[ ]	[x]	Eukaryotic cell lines
[x]	[ ]	Palaeontology and archaeology
[ ]	[x]	Animals and other organisms
[x]	[ ]	Clinical data
[x]	[ ]	Dual use research of concern

Methods

n/a	Involved in the study	
[x]	[ ]	ChIP-seq
[ ]	[x]	Flow cytometry
[x]	[ ]	MRI-based neuroimaging

Antibodies

Antibodies used

APC/Cy7 anti-mouse ITGB1 (clone HMβ1-1) BioLegend Cat# 102226, RRID: AB_2128076 1:300
AF700 anti-mouse CD45.2 (clone 104) BioLegend Cat# 109822, RRID: AB_493731 1:100
FITC anti-mouse CD45.2 (clone 104) BioLegend Cat# 109806, RRID: AB_313443 1:200
FITC anti-mouse BST1 (clone KT157) eBioscience Cat# MA5-17948, RRID: AB_2539332 1:50
APC anti-mouse BST1 (clone BP-3) BioLegend Cat# 140208, RRID: AB_10901172 1:1000
PE anti-mouse PDGFRa (clone APA5) BioLegend Cat# 135906, RRID: AB_1953269 1:100
APC anti-mouse PDGFRa (clone APA5) BioLegend Cat# 135908, RRID: AB_2043970 1:50
PE anti-mouse PDGFRb (clone APB5) BioLegend Cat# 136006, RRID: AB_1953271 1:50
PE/Cy7 anti-mouse CD146 (clone ME-9F1) BioLegend Cat# 134714, RRID: AB_2563109 1:400
AF647 anti-mouse CD146 (clone ME-9F1) BioLegend Cat# 134717, RRID: AB_2721426 1:400
BV510 anti-mouse Ly6C (clone HK1.4) BioLegend Cat# 128033, RRID: AB_2562351 1:200
PE/Cy7 anti-mouse Ly6C (clone HK1.4) BioLegend Cat# 128018, RRID: AB_1732082 1:400
BV421 anti-mouse CD31 (clone MEC13.3) BD Biosciences Cat# 562939, RRID: AB_2665476 1:100 (SPL), otherwise 1:400
PE/Cy7 anti-mouse CD31 (clone MEC13.3) BioLegend Cat# 102524, RRID: AB_2572182 1:100 (SPL), otherwise 1:400
PE anti-mouse VCAM1 (clone 429 (MVCAM.A)) BioLegend Cat# 105714, RRID: AB_1134164 1:100
PE/Cy7 anti-mouse F4/80 (clone BM8) BioLegend Cat# 123113, RRID: AB_893490 1:200
APC anti-mouse F4/80 (clone BM8) BioLegend Cat# 123115, RRID: AB_893493 1:200
APC/Cy7 anti-mouse EpCAM (clone G8.8) BioLegend Cat# 118218, RRID: AB_2098648 1:100
PerCP/Cy5.5 anti-mouse Ly6G (clone 1A8) BioLegend Cat# 127616, RRID: AB_1877271 1:200
PerCP/Cy5.5 anti-mouse Ly6C (clone HK1.4) BioLegend Cat# 128012, RRID: AB_1659241 1:200
PerCP/Cy5.5 anti-mouse Siglec-F (clone S17007L) BioLegend Cat# 155526, RRID: AB_2890714 1:200
PerCP/Cy5.5 anti-mouse TER-119 (clone TER-119) BioLegend Cat# 116228, RRID: AB_893636 1:100
PE anti-mouse Siglec-F (clone S17007L) BioLegend Cat# 155505, RRID: AB_2750234 1:400
BV510 anti-mouse/human CD11b (clone M1/70) BioLegend Cat# 101263, RRID: AB_2629529 1:200
PE/Cy7 anti-mouse CD64 (clone X54-5/7.1) BioLegend Cat# 139314, RRID: AB_2563904 1:200
BV510 anti-mouse CD11c (clone N418) BioLegend Cat# 117353, RRID: AB_2686978 1:200
PE anti-mouse CD115 (clone AFS98) BD Biosciences Cat# 566839, RRID: AB_2869896 1:100
BV785 anti-mouse Ly6C (clone HK1.4) BioLegend Cat# 128041, RRID: AB_2565852 1:500
APC anti-mouse CD11c (clone N418) BioLegend Cat# 117309, RRID: AB_313778 1:200
BV510 anti-mouse I-A/I-E (clone M5/114.15.2) BioLegend Cat# 107636, RRID: AB_2734168 1:200
FITC anti-mouse/human CD11b (clone M1/70) BioLegend Cat# 101205, RRID: AB_312788 1:200
PE/Cy7 anti-mouse TCRb chain (clone H57-597) BioLegend Cat# 109221, RRID: AB_893627 1:200
PE/Cy7 anti-mouse TCRgd (clone GL3) BioLegend Cat# 118123, RRID: AB_11203530 1:200
PE/Cy7 anti-mouse IL-7Ra (clone S18006K) BioLegend Cat# 158209, RRID: AB_2922489 1:200
PE/Cy7 anti-mouse/human CD45R/B220 (clone RA3-6B2) BioLegend Cat# 103221, RRID: AB_313004 1:200
PE/Cy7 anti-mouse NK-1.1 (clone PK136) BioLegend Cat# 108713, RRID: AB_389363 1:200
PE/Cy7 anti-mouse Ly6G (clone 1A8) BioLegend Cat# 127618, RRID: AB_1877261 1:200
Anti-m123/IE1 (MCMV) antibody (clone IE1.01) CapRi Cat# HR-MCMV-12, RRID: unknown 1:200
AF647 anti-mouse IgG (H+L), F(ab')2 Fragment Cell Signaling Cat# 4410S, RRID: unknown 1:500

Validation

All antibodies were obtained from commercial vendors. Validation of individual antibodies can be accessed via the following links:
APC/Cy7 anti-mouse ITGB1 (clone HMβ1-1) https://www.biolegend.com/en-us/products/apc-cyanine7-anti-mouse-rat-cd29-antibody-6184
AF700 anti-mouse CD45.2 (clone 104) https://www.biolegend.com/en-us/products/alexa-fluor-700-anti-mouse-cd45-2-antibody-3393
FITC anti-mouse CD45.2 (clone 104) https://www.biolegend.com/en-us/products/fitc-anti-mouse-cd45-2-antibody-6
FITC anti-mouse BST1 (clone KT157) https://www.thermofisher.com/antibody/product/BST-1-Antibody-clone-KT157-Monoclonal/MA5-17948
APC anti-mouse BST1 (clone BP-3) https://www.biolegend.com/en-us/products/apc-anti-mouse-cd157-bst-1-antibody-7272
PE anti-mouse PDGFRa (clone APA5) https://www.biolegend.com/en-us/products/pe-anti-mouse-cd140a-antibody-6253
APC anti-mouse PDGFRa (clone APA5) https://www.biolegend.com/en-us/products/apc-anti-mouse-cd140a-antibody-6439
PE anti-mouse PDGFRb (clone APB5) https://www.biolegend.com/en-us/products/pe-anti-mouse-cd140b-antibody-6256
PE/Cy7 anti-mouse CD146 (clone ME-9F1) https://www.biolegend.com/en-us/products/pe-cyanine7-anti-mouse-cd146-antibody-9322

AF647 anti-mouse CD146 (clone ME-9F1) https://www.biolegend.com/en-us/products/alexa-fluor-647-anti-mouse-cd146-antibody-15112
BV510 anti-mouse Ly6C (clone HK1.4) https://www.biolegend.com/en-us/products/brilliant-violet-510-anti-mouse-ly-6c-antibody-8726
PE/Cy7 anti-mouse Ly6C (clone HK1.4) https://www.biolegend.com/en-us/products/pe-cyanine7-anti-mouse-ly-6c-antibody-6063
BV421 anti-mouse CD31 (clone MEC13.3) https://www.bdbiosciences.com/en-at/products/reagents/flow-cytometry-reagents/research-reagents/single-color-antibodies-ruo/bv421-rat-anti-mouse-cd31.562939
PE/Cy7 anti-mouse CD31 (clone MEC13.3) https://www.biolegend.com/en-us/products/pe-cyanine7-anti-mouse-cd31-antibody-12996
PE anti-mouse VCAM1 (clone 429 (MVCAM.A)) https://www.biolegend.com/en-us/products/pe-anti-mouse-cd106-antibody-4798
PE/Cy7 anti-mouse F4/80 (clone BM8) https://www.biolegend.com/en-us/products/pe-cyanine7-anti-mouse-f4-80-antibody-4070
APC anti-mouse F4/80 (clone BM8) https://www.biolegend.com/en-us/products/apc-anti-mouse-f4-80-antibody-4071
APC/Cy7 anti-mouse EpCAM (clone G8.8) https://www.biolegend.com/en-us/products/apc-cyanine7-anti-mouse-cd326-ep-cam-antibody-5577
PerCP/Cy5.5 anti-mouse Ly6G (clone 1A8) https://www.biolegend.com/en-us/products/percp-cyanine5-5-anti-mouse-ly-6g-antibody-6116
PerCP/Cy5.5 anti-mouse Ly6C (clone HK1.4) https://www.biolegend.com/en-us/products/percp-cyanine5-5-anti-mouse-ly-6c-antibody-5967
PerCP/Cy5.5 anti-mouse Siglec-F (clone S17007L) https://www.biolegend.com/en-us/products/percp-cyanine5-5-anti-mouse-cd170-siglec-f-antibody-20499
PerCP/Cy5.5 anti-mouse TER-119 (clone TER-119) https://www.biolegend.com/en-us/products/percp-cyanine5-5-anti-mouse-ter-119-erythroid-cells-antibody-4292
PE anti-mouse Siglec-F (clone S17007L) https://www.biolegend.com/en-us/products/pe-anti-mouse-cd170-siglec-f-antibody-16372
BV510 anti-mouse/human CD11b (clone M1/70) https://www.biolegend.com/en-us/products/brilliant-violet-510-anti-mouse-human-cd11b-antibody-7993
PE/Cy7 anti-mouse CD64 (clone X54-5/7.1) https://www.biolegend.com/en-us/products/pe-cyanine7-anti-mouse-cd64-fcgammari-antibody-10062
BV510 anti-mouse CD11c (clone N418) https://www.biolegend.com/en-us/products/brilliant-violet-510-anti-mouse-cd11c-antibody-8491
PE anti-mouse CD115 (clone AFS98) https://www.bdbiosciences.com/en-at/products/reagents/flow-cytometry-reagents/research-reagents/single-color-antibodies-ruo/pe-rat-anti-mouse-cd115-csf-1r.566839
BV785 anti-mouse Ly6C (clone HK1.4) https://www.biolegend.com/en-us/products/brilliant-violet-785-anti-mouse-ly-6c-antibody-11982
APC anti-mouse CD11c (clone N418) https://www.biolegend.com/en-us/products/apc-anti-mouse-cd11c-antibody-1813
BV510 anti-mouse I-A/I-E (clone M5/114.15.2) https://www.biolegend.com/en-us/products/brilliant-violet-510-anti-mouse-i-a-i-e-antibody-7997
FITC anti-mouse/human CD11b (clone M1/70) https://www.biolegend.com/en-us/products/fitc-anti-mouse-human-cd11b-antibody-347
PE/Cy7 anti-mouse TCRb chain (clone H57-597) https://www.biolegend.com/en-us/products/pe-cyanine7-anti-mouse-tcr-beta-chain-antibody-4144
PE/Cy7 anti-mouse TCRgd (clone GL3) https://www.biolegend.com/en-us/products/pe-cyanine7-anti-mouse-tcr-gamma-delta-antibody-7822
PE/Cy7 anti-mouse IL-7Ra (clone S18006K) https://www.biolegend.com/en-us/products/pe-cyanine7-anti-mouse-cd127-il-7ra-antibody-22081
PE/Cy7 anti-mouse/human CD45R/B220 (clone RA3-6B2) https://www.biolegend.com/en-us/products/pe-cyanine7-anti-mouse-human-cd45r-b220-antibody-1930
PE/Cy7 anti-mouse NK-1.1 (clone PK136) https://www.biolegend.com/en-us/products/pe-cyanine7-anti-mouse-nk-1-1-antibody-2840
PE/Cy7 anti-mouse Ly6G (clone 1A8) https://www.biolegend.com/en-us/products/pe-cyanine7-anti-mouse-ly-6g-antibody-6139
Anti-m123/IE1 (MCMV) antibody (clone IE1.01) https://products.capri.com.hr/product/anti-m123-ie1-mcmv-2/
AF647 anti-mouse IgG (H+L), F(ab')2 Fragment https://www.cellsignal.com/products/secondary-antibodies/anti-mouse-igg-h-l-f-ab-2-fragment-alexa-fluor-647-conjugate/4410

Eukaryotic cell lines

Policy information about cell lines and Sex and Gender in Research

Cell line source(s)	Two fibroblast cell lines (NIH3T3 (ATCC Cat. No. CRL-1658) and M2-10B4 (ATCC Cat. No. CRL-1972) were used solely for the purpose of growing virus stocks.
Authentication	Cell lines were obtained from an established public repository (ATCC) with certificates of authentication and were further confirmed by morphological assessment via microscopy upon delivery.
Mycoplasma contamination	Cell lines were tested negative for mycoplasma contamination.
Commonly misidentified lines (See ICLAC register)	Commonly misidentified cell lines were not used in the study.

Animals and other research organisms

Policy information about studies involving animals; ARRIVE guidelines recommended for reporting animal research, and Sex and Gender in Research

Laboratory animals	C57BL/6JrJ mice were purchased from Janvier Labs. B6.129S-Pdgfratm1.1(cre/ERT2)Blh/J mice on C57BL/6J background were purchased from the Jackson Laboratory (IMSR_JAX:032770) and used in comparison to littermate controls. Stat1-/- mice on C57BL/6N background and C57BL/6N controls were kindly provided by Birgit Strobl and Mathias Müller, University of Veterinary Medicine Vienna, Vienna, Austria. Mice were maintained on a 12-hour light and dark cycle at 21°C and 55 % humidity. The study used female mice. Mice were infected at 8-10 weeks of age, except in Fig. 6 where the infection was done at 14 weeks of age.
Wild animals	The study did not involve wild animals.
Reporting on sex	In line with numerous previous reports on MCMV, female mice were used due to the cytotoxicity of salivary gland homogenates from male mice, which impairs virus titrations from this organ.
Field-collected samples	The study did not involve samples collected from the field.
Ethics oversight	Animal procedures were approved as due by The Lower Saxony State Office of Consumer Protection and Food Safety, Germany; by the Ethics Committee at the Faculty of Medicine, Rijeka and Ethics Committee of the Veterinary Department of the Ministry of Agriculture, Croatia, and by the Ethics and Animal Welfare Committee of the University of Veterinary Medicine Vienna and the Austrian Federal Ministry of Science and Research.

Note that full information on the approval of the study protocol must also be provided in the manuscript.

Flow Cytometry

Plots

Confirm that:

- [x] The axis labels state the marker and fluorochrome used (e.g. CD4-FITC).
- [x] The axis scales are clearly visible. Include numbers along axes only for bottom left plot of group (a 'group' is an analysis of identical markers).
- [x] All plots are contour plots with outliers or pseudocolor plots.
- [x] A numerical value for number of cells or percentage (with statistics) is provided.

Methodology

Sample preparation	Peri-gonadal VAT was digested with collagenase P (0.4 mg/ml), dispase II (2 mg/ml) and DNase I (50 µg/ml) in high-glucose GlutaMAX-supplemented DMEM with 10 mM HEPES and 4% fatty acid-free BSA (Sigma-Aldrich) for 55 min at 37°C. The suspension was passed through a 100 µm nylon mesh filter (BD Biosciences), and the floating adipocyte fraction was removed by centrifugation at 400 × g for 6 min. Spleen, LNs and SGs were digested with collagenase P (0.4 mg/ml), dispase II (2 mg/ml) and DNase I (50 µg/ml) for 30 min at 37 °C and then again for additional 20 min, followed by incubation with 5 mM EDTA for 5 min. CD45- cell fractions from the spleen and LNs were enriched by depletion of CD45+ cells while splenic macrophages were enriched by positive selection of VCAM-1+ cells using MACS (Miltenyi Biotech). Lungs were injected with 2.5 ml digestion solution containing collagenase P (0.4 mg/ml), dispase II (2 mg/ml) and DNase I (50 µg/ml), minced with scissors and then processed in gentleMACS Dissociator (Miltenyi Biotech) using program "37C_m_LDK_1". Liver digestion was performed using the retrograde perfusion technique via the inferior vena cava described by Mederacke et al. with modifications. Briefly, organs were in situ perfused with 10 ml of pre-warmed Liver Perfusion Medium (Gibco) followed by injection of 20 ml of pre-warmed digestion medium (1 mg/ml collagenase P, 1 mg/ml dispase II, 50 µg/ml DNase I in Gibco Hank's Balanced Salt Solution (HBSS) with 10 mM HEPES and 1.5 mM calcium chloride) over 5-7 min. Afterwards, organs were dissected, minced with scissors and in vitro digested for 45 min at 37°C. Non-parenchymal cells were enriched by two rounds of centrifugation at 50 × g for 5 min whereupon only supernatant was collected.
Instrument	Data acquisition was performed on an Aria-II SORP, ARIA-Fusion or LSR-Fortessa (BD Biosciences).<br>Sorting was performed on an Aria-II SORP or ARIA-Fusion (BD Biosciences).
Software	FlowJo software 10 (BD)
Cell population abundance	Sorting purities were routinely determined by flow cytometric re-analysis of sorted cell populations. Samples showing ≥ 95% purity of sorted cells were qualified for downstream analysis.
Gating strategy	All flow cytometric analyses were performed following exclusion of dead cells (identified using 7-AAD Viability Staining Solution or Zombie NIR or Zombie Violet Fixable Viability Kits; all from BioLegend) and cell aggregates (identified on FSC-A versus FSC-H scatter plots). Gating strategy and precise marker definition for all populations studied are shown in Figure 1, Figure 3, Supplementary Figure 1 and Supplementary Figure 2.

- [x] Tick this box to confirm that a figure exemplifying the gating strategy is provided in the Supplementary Information.
